# Leveraging genome-scale metabolic models to understand aerobic methanotrophs

**DOI:** 10.1093/ismejo/wrae102

**Published:** 2024-06-11

**Authors:** Magdalena Wutkowska, Vojtěch Tláskal, Sergio Bordel, Lisa Y Stein, Justus Amuche Nweze, Anne Daebeler

**Affiliations:** Institute of Soil Biology and Biogeochemistry, Biology Centre CAS, 370 05 České Budějovice, Czech Republic; Institute of Soil Biology and Biogeochemistry, Biology Centre CAS, 370 05 České Budějovice, Czech Republic; Department of Chemical Engineering and Environmental Technology, School of Industrial Engineering, University of Valladolid, Valladolid 47011, Spain; Institute of Sustainable Processes, Valladolid 47011, Spain; Department of Biological Sciences, Faculty of Science, University of Alberta, Edmonton, AB T6G 2E9, Canada; Institute of Soil Biology and Biogeochemistry, Biology Centre CAS, 370 05 České Budějovice, Czech Republic; Department of Ecosystem Biology, Faculty of Science, University of South Bohemia, 370 05 České Budějovice, Czech Republic; Department of Science Laboratory Technology, Faculty of Physical Sciences, University of Nigeria, Nsukka 410001, Nigeria; Institute of Soil Biology and Biogeochemistry, Biology Centre CAS, 370 05 České Budějovice, Czech Republic

**Keywords:** metabolic modelling, methane oxidisers, systems biology

## Abstract

Genome-scale metabolic models (GEMs) are valuable tools serving systems biology and metabolic engineering. However, GEMs are still an underestimated tool in informing microbial ecology. Since their first application for aerobic gammaproteobacterial methane oxidizers less than a decade ago, GEMs have substantially increased our understanding of the metabolism of methanotrophs, a microbial guild of high relevance for the natural and biotechnological mitigation of methane efflux to the atmosphere. Particularly, GEMs helped to elucidate critical metabolic and regulatory pathways of several methanotrophic strains, predicted microbial responses to environmental perturbations, and were used to model metabolic interactions in cocultures. Here, we conducted a systematic review of GEMs exploring aerobic methanotrophy, summarizing recent advances, pointing out weaknesses, and drawing out probable future uses of GEMs to improve our understanding of the ecology of methane oxidizers. We also focus on their potential to unravel causes and consequences when studying interactions of methane-oxidizing bacteria with other methanotrophs or members of microbial communities in general. This review aims to bridge the gap between applied sciences and microbial ecology research on methane oxidizers as model organisms and to provide an outlook for future studies.

## Introduction

Genome-scale metabolic models (GEMs, sometimes abbreviated as GSMMs) are widely used in systems biology to quantitatively link genomic information with the phenotype of a cell [1, 2]. Depending on the underlying aims, modelling assumptions, nature of input data, and desired outcomes, metabolic models can be established under three modelling frameworks: dynamic, constraint-based, or a hybrid of the two [3, 4]. Dynamic methods reconstruct metabolism, taking into account the kinetics of metabolic reactions. Moreover, they can be used to calculate the change in metabolite concentration over time [3, 5]. In the absence of kinetic data at the genome level and the availability of annotated genomes, constraint-based methods reconstruct metabolism in a steady state. Most available GEMs are constraint-based models [6] using flux-balance analysis (FBA). FBA mathematically describes how metabolites flow through the system in a steady state based on a matrix of metabolites, metabolic reactions, and stoichiometry [7]. In this matrix, the number of reactions is higher than the number of metabolites. Therefore, there are many possible ways in which these flows can be solved. Depending on the problem, the model aims to predict a chosen phenotype within the defined conditions and constraints, typically related to growth. Defining this resulting outcome as an equation (objective function) shrinks the space of possible solutions to the optimal one that maximizes the phenotype [7]. As a result, the model quantifies the contribution of each reaction to the phenotype. Finally, hybrid GEMs use some aspects of both frameworks, e.g. [4]. Even though there have been studies utilizing the dynamic framework in the context of methanotrophs, most metabolic modelling efforts conducted for this microbial group have used the constraint-based framework.

The application of GEMs in microbial research is rapidly growing and ranges from single-strain metabolic network analyses and engineering to the prediction of complex community dynamics [13–16]. The main steps and best practices of GEM reconstruction have been summarised earlier; however, they are mostly connected with constraint-based methods and are for single species only [14, 17–21]. In these cases, the model construction is an iterative process in which discrepancies between predictions and experimental data are used to correct previous model versions. Thereby, GEMs complement phenotypic data with quantitative insights into the metabolic reactions taking place in the system under defined conditions.

Increased interest in applying GEMs to various problems in microbial engineering and systems biology has resulted in the development of new tools and approaches to analyze GEMs of single strains and microbial communities [13, 16, 22–27]. However, we believe that the full potential of GEMs for microbial ecology, especially in an environmental context, has not yet been unlocked. By highlighting knowledge advances for aerobic methanotrophic bacteria through GEM applications, we want to inspire and encourage the wider use and further improvement of this tool within the larger field of microbial ecology. However, in this review, we focus on methanotrophs as they represent keystone ecological guilds in many habitats and possess high industrial potential. Methanotrophs that perform aerobic methane oxidation can convert methane produced within an ecosystem (e.g., wetlands, lake sediments) or methane diffused to the atmosphere and utilise it as a carbon and/or energy source. They are members of the *Alpha-* and *Gammaproteobacteria*, *Verrucomicrobiae*, *Actinomycetia*, and *Methylomirabilia*, relying either on particulate or soluble methane monooxygenases (pMMO and sMMO, respectively) for the first step of methane oxidation in a broad range of ecological niches [28–38].

In this review, we first collected all studies published to date on researching aerobic methanotrophs using GEMs (Table 1; see Table 1 caption for the methodological details). We then selected areas of aerobic methanotroph ecology that have benefited most from applying GEMs and summarized the accomplished knowledge advances. In addition, we defined methodological challenges when applying GEMs to aerobic methanotrophs and identified promising directions for their future applications.

**Table 1:** Publications reporting on employment of GEMs for exploring aerobic methanotrophs.

	Main focus of the GEM	*-omics*	Method	Ref
One-strain GEMS				
***Methylo(tuvi)microbium***				
*M. buryatense* 5GB1	Source of electrons for pMMO	T	FBA	[42]
*M. buryatense* 5GB1C	Response to oxygen limitation	T	FBA	[63]
*M. buryatense* 5GB1	Production of fatty acids	T	FBA	[86]
*M. buryatense* 5GB1	Metabolism using different C1 substrates	T, M	FBA	[69]
*M. buryatense* 5GB1C	Metabolism using different C1 substrates under limiting conditions	M	FBA	[127]
*M. buryatense* 5GB1	Oxygen- and methane-limited phenotypes	T^*^	FBA	[128]
*M. buryatense* 5GB1C	Entner–Doudoroff pathway	-	FBA	[129]
*M. buryatense* 5GB1C	Metabolism at different methane concentrations	T	FBA	[79]
*M. alcaliphilum* 20ZR	Reconstruction of core metabolism	M	FBA	[53]
*M. alcaliphilum* 20ZR	Role of rare earth elements in physiology	T, P, M	FBA	[130]
*M. alcaliphilum* 20Z	Production of 2,3-butanediol	-	FBA, OptGene	[131]
*M. alcaliphilum* 20ZR	Production of muconic acid	-	FBA	[132]
*M. alcaliphilum* 20Z	Production of putrescine	T	FBA, OptGene	[133]
*M. alcaliphilum* 20Z	Metabolism using different C1 substrates	T, M	FBA	[70]
*M. alcaliphilum* 20Z	Regulation of C1 metabolism	T	FBA	[134]
*M. alcaliphilum* 20Z	Halotolerance mechanism; production of ectoine	T	FBA	[90]
*M. alcaliphilum* 20Z	Production of α-humulene, α-bisabolene	T^*^, M^*^	FBA	[135]
*M. alcaliphilum* 20Z (engineered)	Production of shinorine, 2,3-butanediol, acetoin, and 3-hydrobutyric acid from methane and xylose	T	FBA	[136]
*M. album* BG8	Production of biomass and organic compounds	T^*^, M^*^	FBA	[12]
***Methylococcus***				
*M. capsulatus* (Bath)	Source of electrons for pMMO	-	FBA	[54]
*M. capsulatus* (Bath)	Entner–Doudoroff pathway	-	FBA, FVA	[8]
***Methylocystis***				
*M. parvus* OBBP	Source of electrons for pMMO, production of poly-3-hydroxybutyrate	-	FBA	[56]

*M. hirsuta* CSC1	Source of electrons for pMMO; production of poly-3-hydroxybutyrate	-	FBA	[55]
*M.* sp.SC2	Source of electrons for pMMO; production of poly-3-hydroxybutyrate	-	FBA	[55]
*M.* sp. SB2	Source of electrons for pMMO; production of poly-3-hydroxybutyrate	-	FBA	[55]
***Methylosinus***				
*M. trichosporium* OB3b	Source of electrons for pMMO	-	FBA	[57]
*M. trichosporium* OB3b	Production of cadaverine	-	FBA, FVA	[11]
*M. trichosporium* OB3b	Production of 2-hydroxyisobutyric acid and 1,3-butanediol	T	FBA, FVA	[10]
***Methylocella***				
*M. silvestris* BL2	Metabolism of different C1 and C2 substrates	P	FBA	[59]
***Methylacidiphilum***				
*M. fumariolicum* Pic	Reconstruction of methano-, auto-, and heterotrophic metabolism	T^*^	FBA	[78]
**Two-strain GEMs**				
*Methylobacter tundripaludum* 21/22 and *Methylomonas* sp. LW13	Interactions under resource limiting conditions	T^*^	FBA, FVA	[9]
Methanotroph—phototroph coculture (*M. buryatense* and *Arthrospira platensis*)	Interactions to enhance biogas conversion	-	FBA, dFBA	[81]
**Community GEMs**				
Chemolithoautotrophic community	Competition and cooperation at an ecosystem level		MRO, MIR	[137]

The table is sorted by organisms and highlights the main research focus of each publication, whether *-omics* data were included in the study, and the reported methods used. Publications for this systematic review were found using the following search string in Scopus: “TITLE-ABS-KEY-AUTH (“genome-scale metabolic model” OR “genome scale metabolic model” OR “metabolic model”) AND TITLE-ABS-KEY-AUTH (“methane” OR “methane oxidation” OR “methane oxidiser*” OR “methanotrophs”)”. Only publications fulfilling the following two criteria were included: using genome-scale metabolic models (I) and exploring methane transformations in known aerobic methanotrophs (II). Additionally, we screened all the referenced papers and papers citing the publications to fulfil both criteria. Abbreviations: T, transcriptomics; M, metabolomics; P, proteomics; *, data taken from other studies; FBA, flux balance analysis; dFBA, dynamic flux balance analysis; FVA, flux variance analysis; OptGene, a method used to predict gene knockout targets; MRO, metabolic resource overlap; MIR, metabolic interaction potential.

## Better understanding of aerobic methanotrophs through GEMs

**Figure 1:**
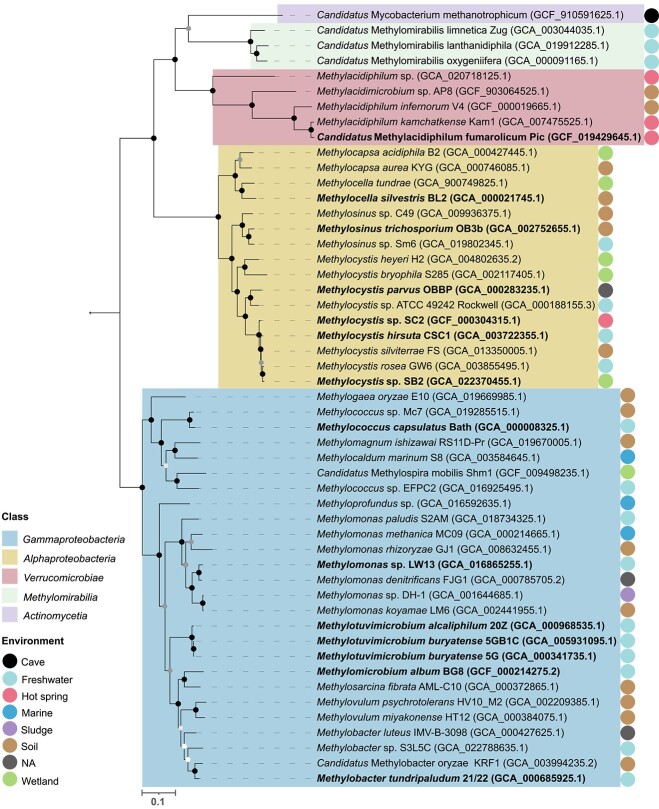
Phylogenetic diversity of methanotrophs that perform aerobic methane oxidation. Names in bold indicate strains that have a reconstructed GEM. The tree, based on a concatenated protein alignment of 71 marker genes (Supplementary S1), was created using anvi’o [138] with third-party software: HMMER [139], Prodigal [140], and MUSCLE [141] as sequence aligner. The phylogenetic tree was generated using IQ-TREE with a maximum likelihood approach and the WAG model with 1000 bootstrap iterations [142, 143]. The genome of *Nitrosomonas europaea* ATCC 19718 (GCA_000009145) was used as an outgroup. Microbial genomes and associated metadata were obtained from the NCBI (https://www.ncbi.nlm.nih.gov/data-hub/genome/). Black, dark grey, light grey, and white circles at nodes denote 100%, >90%, >70%, and > 50% bootstrap support, respectively. Organisms for which a GEM has been constructed are highlighted in bold. Colored circles indicate the source of a sequenced genome.

**Figure 2:**
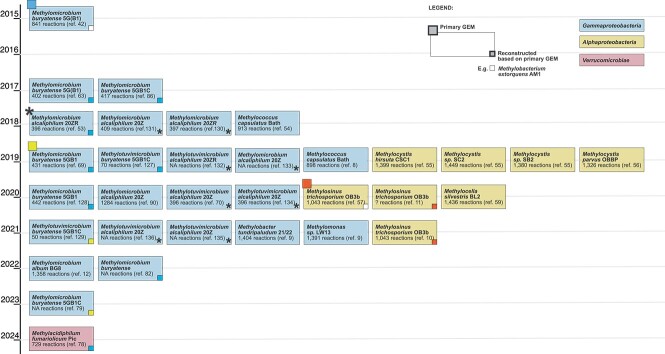
Genealogy of methanotroph GEM models. The diagram shows the evolution and genealogy of GEMs of methanotrophic microorganisms since the reconstruction of the GEM for *Methylomicrobium buryatense* 5GB1 [42]. Each box represents a single metabolic model and includes the species name and the number of reactions incorporated (NA indicates that the article or supplementary materials did not specify the number of reactions.) Two GEMs with a white square in the lower right corner were reconstructed based on a GEM of the methylotroph *Methylobacterium extorquens* AM1 [144].

The first GEMs were reconstructed in 1999 and the early 2000s [39–41], yet the first manually curated model of a methanotroph exploring the metabolism of *Methylomicrobium buryatense* 5G(B1) was introduced only in 2015 [42]. Nearly a decade later, GEMs have been reconstructed for 13 aerobic methanotrophs belonging to *Alphaproteobacteria*, *Gammaproteobacteria,* and *Verrucomicrobiae* (Figs 1 and 2, Table 1) and analyzed predominantly only with FBA (Table 1). Another constraint-based method, flux variability analysis (FVA), has been used less commonly [8–12]. However, it provides useful information on the range of reaction flux values that satisfy the FBA problem. Besides reaching the overarching aim of genome reconstruction and inspecting carbon fluxes under different conditions, many of the GEM studies explored long-standing questions about methanotrophic metabolism, the production of various metabolites by methanotrophs, and some started to disentangle the interactions of methanotrophs with other organisms (Table 1). In the forthcoming sections, we focus on these selected topics due to their relevance in the field of microbial ecology, while other reviews highlighting the usage of GEMs for methanotrophs in the field of microbial engineering have been provided elsewhere [43–48].

### The great electron quests

One of the knowledge gains in aerobic methanotroph metabolism, to which GEMs have substantially contributed, is a more refined understanding of the source of electrons needed to reduce oxygen to water by the methane-oxidizing enzyme pMMO. Unlike in the case of electron transfer from NADH to the sMMO [49], the source(s) of electrons for the pMMO catalyzed reaction had remained a crucial knowledge gap in the field [50]. Especially because most known aerobic methanotrophs rely only on pMMO [51], this gap restricted our complete understanding of the rate-limiting step of methane oxidation in general. Three possible scenarios of electron sources had been identified, but their realization, if at all, remained highly uncertain. In the *redox arm* scenario, ubiquinol would be generated from NADH via complex I of the electron transport chain and drive methane oxidation, while electrons from methanol oxidation would be used for ATP production. In the *direct coupling* scenario, electrons generated from methanol oxidation would directly be transferred to pMMO for methane oxidation. Finally, in the *uphill electron transfer* scenario, electrons from methanol oxidation would partially drive methane oxidation by feeding back to the ubiquinol pool via a reverse electron flow (see [52] for a current overview).

Applying GEMs to this knowledge gap has led to a more refined understanding and identified conditions under which the operation of the proposed scenarios for pMMO reduction are most likely in different methanotrophs. More precisely, in all studies highlighted below, the authors carefully compared model predictions for each of the three scenarios with the empirical data obtained from pure cultures. Doing so, they found that some gammaproteobacterial methanotrophs, such as *M. buryatense* 5G(B1) and *Methylomicrobium alcaliphilum* 20Z^R^, both assimilating carbon through the ribulose monophosphate pathway (RuMP), are most likely using the *direct coupling* mode of pMMO reduction [42, 53]. Other methanotrophs like *Methylococcus capsulatus* (Bath), using RuMP and possessing enzymes for the Calvin-Benson-Bessham cycle, may change from *direct coupling* to *uphill electron transfer* when the oxygen-to-methane ratio increases beyond the point that allows for maximum growth [54]. In contrast, methanotrophs using the serine cycle for carbon assimilation, such as *Methylocystis* and *Methylosinus,* are more likely to employ the *redox arm* mechanism [55–57]. The utilization of this less efficient mode of pMMO reduction, which couples methane oxidation with complex I of the respiratory chain, matches the typically lower biomass yield of these methanotrophs [56, 58].

### C1 assimilation and anaplerotic reactions

Aerobic methanotrophs use two main pathways to assimilate carbon from methane: the serine cycle or the RuMP cycle. Intermediates of both cycles are used to synthesize biomass building blocks. They are also needed to feed into the tricarboxylic acid (TCA) cycle, which generates NADH to obtain energy in the respiratory chain. Anaplerotic reactions are therefore needed to replenish the TCA or serine cycle to balance the drainage of intermediates for biomass synthesis. Several studies leveraging GEMs have addressed this topic within the last decade. First, an investigation of three members of the genus *Methylocystis* (*M. hirsuta* CSC1, *M.* sp. SC2, and *M.* sp*.* SB2) confirmed the necessity of glycine synthase for replenishment of the serine cycle during cell growth. After carboxylation of 5-10-methylenetetrahydrofolate (with concomitant consumption of NADH and ammonium), the produced glycine can enter the serine cycle, which is connected to the TCA cycle by sharing oxaloacetate and malate [55]. Secondly, another GEM study confirmed that *Methylocella silvestris*, which is able to grow on C1 and C2 compounds [29], relies on the glyoxylate shunt (formed by isocitrate lyase and malate synthase) in order to replenish the serine and TCA cycles when growing on C2 compounds [59]. The study furthermore found that *M. silvestris* relies on glyoxylate produced by isocitrate lyase to replenish the TCA cycle via the serine cycle, in which glyoxylate is an intermediate when growing on C1 substrates. Thus, the serine cycle in *Methylocella* is replenished at a different entry point than in *Methylocystis.* Yet another mode of anaplerotic replenishment of TCA cycle intermediates was quantified via FBA in a GEM for *Methylocystis parvus* OBBP [56]. This alphaproteobacterial methanotroph was known to synthesize and store polyhydroxybutyrate (PHB) during nitrogen-limiting conditions. Once nitrogen is replenished, cells metabolize the stored PHB but can only grow if methane is available [60]. The GEM of *M. parvus* included PHB degradation with an anaplerotic role by supplying glyoxylate to the serine cycle and succinyl-CoA to the TCA cycle [56]. The model predicted a three-fold decrease of dependence on glycine synthase for anaplerotic reactions when both PHB and methane were co-metabolized compared to sole methane metabolism due to the anaplerotic role of PHB degradation.

In regards to carbon assimilation, a GEM study incorporating global metabolomics profiles and enzymatic assays in the gammaproteobacterial methanotroph *M. alcaliphilum* 20Z^R^ demonstrated that four pyrophosphate (PPi)-dependent reactions operate in the central C1 metabolism, instead of analogous ATP-dependent reactions [53]. For instance, *M. alcaliphilum* 20Z^R^ uses the PPi-dependent, instead of the ATP-dependent, 6-phosphofructokinase to save energy as an entry point for glycolysis, and it uses pyruvate phosphate dikinase to catalyze the conversion of phosphoenolpyruvate to pyruvate with the generation of ATP [53]. Furthermore, a highly branched tricarboxylic acid cycle in *M. alcaliphilum* 20Z^R^ with several variants for the conversion of 2-oxoglutarate to succinate along with an aspartate-fumarate loop and a carbon shunt from acetyl-CoA to the RuMP cycle via the phosphoketolase pathway were proposed [53]. Together, these metabolic modifications contribute to energy preservation and improve carbon conversion efficiency.

### Response to substrate shifts

Typically, aerobic methanotrophs inhabit systems that undergo dynamic shifts in redox conditions and chemistry on a scale of minutes to days, seasonally or otherwise, alongside superimposed long-term shifts due to global change. For instance, limiting oxygen availability can be a common reoccurring condition in many ecosystems and is predicted to become even more severe with progressing global change features, such as eutrophication and warming [61, 62]. For aerobic methanotrophs, oxygen depletion leads to decreased methane consumption and increases or alters short-chain organic acid excretion as metabolism shifts from methane respiration to fermentation [63, 64]. The excretion of different organics under fully oxic as opposed to oxygen-limited conditions leads to crossfeeding of distinct microorganisms with methane-derived carbon in natural communities [65, 66]. Moreover, the excretion profiles of even closely related methanotrophic species differ, containing metabolites such as methanol, formate, acetate, succinate, lactate, or other compounds, e.g. hydrogen, and the impacts on their immediate surroundings and any biotic interactions are therefore expected to differ as well [63, 64, 67, 68]. A GEM constructed for *M. buryatense* suggested that the increased excretion of acetate under oxygen-depleted conditions is a response to redox imbalance. At the same time, the model predicted the fraction of ATP generation from substrate-level phosphorylation to be roughly 71%, which provided quantitative insights into the mixed mode of metabolism via simultaneous respiration and fermentation [63]. Likewise, a GEM constructed for *Methylomicrobium album* BG8 predicted that this methanotroph excretes acetate under lower oxygen conditions through a mixed metabolic mode, in which pathways for respiration and fermentation are both active [12]. In the same study, the authors used their experimentally validated GEM to simulate the organism’s system-level metabolic reaction to different oxygen-to-methane ratios. They found that biomass production was optimal at a 1.5 oxygen-to-methane ratio. However, both biomass and ATP production were feasible between a range of >0 to 2.5. The GEM analysis further predicted that mainly formaldehyde oxidation and assimilation in *M. album* BG8 change at different oxygen-to-methane ratios. With a close to optimal oxygen-to-methane ratio, formaldehyde was predicted to be efficiently oxidized through the tetrahydromethanopterin pathway and subsequently assimilated in the RuMP cycle and its Embden-Meyerhof-Parnas (EMP) variant. However, at lower oxygen-to-methane ratios, the folate pathway was predicted to contribute to formaldehyde oxidation and assimilation through the Entner–Doudoroff (ED) variant of the RuMP cycle. At the same time, the excretion of methanol was found to be necessary to maintain efficient methane oxidation under low oxygen levels.

GEMs for aerobic methanotrophs able to utilize C2 compounds, like ethanol, propane, and acetate, have helped determine and quantify the species-specific changes in carbon flux through core carbon metabolic pathways. Furthermore, modelling helped to describe the excretion profiles of small-chain organics when switching to these alternative substrates [8, 59, 69, 70]. During methane metabolism by *M. buryatense* 5GB1, the GEM predicted a ratio of 3:1 of the EMP and ED RuMP cycle variants for formaldehyde assimilation, and an increased relative reliance on the ED variant was predicted under methanol metabolism with a flux ratio of 1:1 [69]. Quite oppositely, a GEM constructed for *M. alcaliphilum* 20Z suggested an increase of relative carbon flow from formaldehyde via the EMP-RuMP cycle by changing the ratio of EMP to ED RuMP from 3:1 to roughly 15:1 when switching from growth on methane to methanol [70]. This discrepancy in methanol metabolism modelled for *M. buryatense* and *M. alcaliphilum* is unexpected because these organisms are closely related. Nonetheless, they seem to differ in their ability to avoid formaldehyde toxicity [71].

Some of the most extremophilic aerobic methanotrophs belonging to *Verrucomicrobiae* exhibit the highest metabolic flexibility among characterized methanotrophs [25, 68–74]. The thermoacidophilic *Methylacidiphilum fumariolicum* switches its metabolism between three substrate scenarios: methanotrophy, auto-, and heterotrophy (on both C2 and C3), radically altering fluxes and the necessary redox trade-offs within the cell [78]. Particularly for methanotrophy and autotrophy, the model predicted a decrease in growth compared to heterotrophy, which was attributed to the use of the different pathways but caused by a similar mechanism—the very high energy cost to replenish redox equivalents.

Isolated methanotrophs are usually cultured under conditions maximizing their growth, which includes headspace methane concentrations ranging from 5–50%. Therefore, the GEMs constructed for them typically do not include methane as a limiting nutrient. However, recent interest in aerobic methanotrophs able to oxidize methane at as low as atmospheric concentrations (i.e. 1.8 ppm) is rapidly growing with the increasing need for efficient climate change mitigation strategies. Genome-scale metabolic modelling was employed to predict the non-growth-associated ATP maintenance energy of *M. buryatense* at methane concentrations ranging from 200 to 1000 ppm, showing that *M. buryatense* is able to lower its needed maintenance energy as a function of decreased methane availability in order to sustain survival [79, 80].

### Interactions of aerobic methanotrophs with other organisms

There is limited knowledge about the dynamics of biotic interactions between methanotrophs and other organisms within the same system. GEMs hold the potential to explore metabolic relationships between cells or species. However, when writing this review, only two studies modelled a two-strain GEM to disentangle interactions between an aerobic methanotroph and another microorganism [9, 81]. In the first study, manually curated GEMs of a simplified methanotrophic community from Lake Washington (represented by *Methylomonas* sp. LW13 and *Methylobacter tundripaludum* 21/22*)* were subsequently incorporated into a community model [9], using the bi-level multiobjective optimization framework OptCom [82]. As the name suggests, this model uses optimization on two levels: for each community member and for the community, with the biomass as an objective function. With this model, the authors investigated limiting inputs of methane, oxygen, and nitrogen to obtain possible scenarios of changes in the endo−/exometabolites and the dynamics in this simplified community. The predictions indicated that in the high-nutrient scenario, *Methylomonas* outcompeted *Methylobacter*, producing twice as much biomass. However, in scenarios where either oxygen, nitrogen, or methane were limited, *Methylobacter* constantly exhibited greater biomass than *Methylomonas*, particularly in methane- and oxygen-limiting conditions. The composition and quantities of excreted molecules were predicted to differ between limiting scenarios; for instance, the highest carbon dioxide production was predicted in the nitrogen-limited condition, with *Methylomonas* emitting nearly four times as much as *Methylobacter*.

Another two-strain GEM study explored the interactions within a biogas-fed coculture of an aerobic methanotroph (*M. buryatense*) and a cyanobacterium (*Arthrospira platensis*) [81] using the Microbiome Modelling Toolbox [83]. Here, the authors combined both constraint-based and hybrid methods to model cell growth and changes in metabolite production. In this coculture, the methanotroph consumed biogas-methane and the oxygen produced by the photoautotroph through photosynthesis, while the photoautotroph consumed biogas-carbon dioxide and the carbon dioxide produced by the methanotroph through methane oxidation [81]*.* The *in silico* analysis identified possible mutual growth-stimulating interactions through the exchange of metabolites, such as succinate, formate, and ammonium, which resulted in a > 40% increase in the optimal growth rate of both species in the coculture when compared to axenic conditions.

The (eco)system-level models of these two studies have defined several conditions with expected phenotypes and pinpointed scenarios under which the dynamics between methanotrophs and other community members are likely to vary. Their predictions serve as ready-made testable hypotheses for laboratory explorations and are, therefore, a valuable tool for better understanding methanotroph ecophysiology and interconnected community interactions.

## Methodological considerations and challenges

There are many tools for the automated creation of draft GEMs [84]; however, manual curation of the initial GEM draft is still highly recommended to obtain a reliable model of a single strain. Curation involves (but is not restricted to) removing reactions producing ATP in a thermodynamically infeasible manner, adding metabolic reactions absent in the draft but evident from experimental data, and correcting wrongly annotated gene-reaction associations.

Unlike GEMs for model organisms like *Escherichia coli* [85], most of those constructed for methane oxidizers are still in the initial stages of the recently proposed GEM life cycle [20]. Thus, they have been constructed for specific species and rarely used subsequently (Fig. 2). However, a few of the first methanotroph GEMs have been iterated, reused, and refined, and their scope and predictive range have been expanded by including additional metabolic pathways, e.g. lipid metabolism [86]. Extending the life cycle of a GEM can be a collective effort of iteration, addition, and development, leading to increased quality so that the GEM can easily be joined with other models to investigate more than one organism at a time [20].

Despite experimental validation being an integral part of analyzing a GEM, e.g. [7, 14, 21], not all the GEM studies reviewed here were experimentally validated. Some rely on validation with data obtained solely from literature. Model quality should be assured with experimental validation followed by changes to the subsequent iterations of that particular model [20]. Additionally, some aspects of GEM quality, such as annotations compliant with current recommendations [87], can be assessed during model construction using software such as MEMOTE [88]. A powerful validation and refinement approach is the integration of *-omics* data, such as transcriptomics, proteomics, or (untargeted) metabolomics, into GEMs, thereby building additional levels of information into its model regulatory networks. Moreover, including -*omics* data and experimentally determined physiological data can be an effective tool for identifying best-fitting metabolic flux distributions and quantifying changes in metabolic fluxes between experimental conditions [89, 90]. However, integrating *-omics* data is not a straightforward task [91]. It requires solving several problems, including the ambiguous connection between flux rates and levels of transcripts or proteins, and dealing with biological noise in the data.

Many aerobic methanotrophic bacteria encode multiple gene clusters for pMMO, and some gammaproteobacterial methanotrophs further encode a sequence-divergent particulate monooxygenase (pXMO) [92–94]. The paralogous gene clusters of pMMO can play different roles, as they have been found to encode low vs. high-affinity methane monooxygenase and are differentially utilized in the presence of low vs. high ammonium concentrations [92, 95]. Likewise, the pXMO enzyme is thought to enable methane oxidation under hypoxia [94, 96]. However, GEM models are essentially blind to such paralogous genes as their pathway function is identical, and differences in their primary sequences are usually not easily detected. As an example of paralog blindness, the GEM for *M. album* BG8 accurately predicted the reactions that contributed to methane oxidation and growth under low oxygen but did not capture the relative contribution of its pMMO versus pXMO paralogs [12]. Thus, if the differential expression of paralogous genes is of interest, these details would need to be analyzed in separate empirical experiments. Currently, none of the available GEMs for aerobic methanotrophs using a hybrid method, such as dynamic FBA, account for the kinetics of paralogous pMMO/pXMO enzymes. Connecting the paralogous gene clusters with variable kinetic properties or differential activities across gradients of substrates could significantly improve the understanding of ecophysiology in dynamic settings.

The advancement of using and improving GEMs for methanotrophs is hindered by data availability. Upon publication of a new GEM, the construction should be made available online in a file format that enables easy access and re-usability of the data independent of the specific tools used for its construction [87, 88]. At the same time, uniformly coded annotations and code for *in silico* analysis need to be provided. Ensuring reproducibility in this way enhances the re-usability of the models but also poses a question about (re-)analyzing data generated with software that relies on paid licensed dependencies like the COBRA Toolbox requiring MATLAB [97] versus cobrapy [98] or R packages such as *sybil* [99], to name a few.

## Future perspectives

To date, GEM studies of aerobic methanotrophs have focused on *Gamma*- and *Alphaproteobacteria* and, most recently, on a *Verrucomicrobiae* methanotroph [78]. Future GEMs could focus on other methanotrophs of this metabolically flexible class [34, 72, 75–77] and on facultative methanotrophs belonging to the *Alphaproteobacteria*. Most aerobic methanotrophs, for which GEMs have been reconstructed, represent promising and efficient platforms to produce value-added products and were mainly isolated from freshwater (Fig. 1, Table 1). Expanding the available repertoire of GEMs for methanotrophs from different environments and widening the focus beyond biotechnological applications holds great potential to further enhance our understanding of these organisms and the constraints that an environment may put on the inhabiting methanotrophs. Finally, methanotrophs exhibit unique suites of biosynthetic capabilities [100–105] with often unknown metabolic roles for their particular phenotypes (e.g. survival under hypoxia and growth on atmospheric methane), which could be further investigated in an ecological context. These unique capabilities could likely be overlooked in automatic reconstructions and, therefore, require careful manual curation.

Beyond the pending construction of GEMs to cover the full taxonomic and metabolic diversity known for aerobic methanotrophs, the reconstruction and analyses of community GEMs, which focus on the interaction of methanotrophs with other (micro)organisms, is a needed and highly promising area of future GEM research. As members of natural microbiomes, methanotrophs interact and “communicate” with diverse organisms, including algae [106–108], mosses, and plants [109–112], marine invertebrates [113], as well as with many heterotrophic microorganisms [114, 115]. The basis for these interactions is often shared metabolites, such as volatiles and short-chain organic acids, that enable interspecies signalling or cross-feeding [67, 116–118]. It could even be demonstrated that the presence of a heterotrophic syntroph can trigger a metabolic switch in a methanotroph, leading to the production of the cross-feeding metabolite methanol [67]. The extent of such metabolic interactions between methanotrophs and other microorganisms has further been shown to depend on the available carbon sources and oxygen levels [66, 119, 120]. Such interactions have been documented in laboratory experiments, but as mentioned above, they have not been a focus of genome-scale modelling. GEMs, with their system-wide prediction, constitute a useful and underused tool to quantify microbial interactions and to unravel their molecular basis and regulation under variable conditions.

High-throughput metabolic reconstruction and modelling efforts, such as those used for human gut microbial genomes [121, 122], might be applied to bioreactor communities or dynamic natural habitats with a high abundance of methanotrophs, such as wetland soils and landfills. These types of studies would provide a starting point for understanding the role of individual microbes, including methanotrophs, within complex communities. GEMs have mainly been applied to relatively simple microbial systems, and there are many challenges when shifting to natural microbial communities, including the increasing complexity of interactions [23]. As human microbiome studies are often at the forefront of developing methods and tools, many advances in community-level GEMs originated from this field. Numerous tools for modelling communities under both constraint-based or dynamic frameworks, as well as many computational pipelines for their analyses, have been developed in the recent decade. Many of these approaches shift towards the automatic construction of GEMs derived from genomes or metagenome-assembled genomes. Although these approaches enable a high-throughput and quick way to obtain draft models, they require a careful evaluation of their quality and perhaps even manual curation, especially when it comes to genes prone to misidentification or those holding unique capabilities. Moreover, even though community-level GEMs allow for the modelling of resource competition between microorganisms, they disregard other types of possible interactions, such as predation or volatile-mediated interactions, which have also been shown to be important for methanotrophs [117, 123]. Some recent developments, however, started to address these issues by accounting for microbial “functional guilds” and the role of spatial heterogeneity in the environment [13, 26, 124].

The scarcity of community GEM studies from ecosystems that produce and consume high volumes of methane is a more urgent need as climate change accelerates. Here, we argue that microbial community models could significantly increase our knowledge of the flux of methane-derived carbon through a system similar to what has been achieved by applying GEMs, e.g., to the phyllosphere [125] and rhizosphere context [126]. The construction of community GEMs targeting habitats that support highly active and abundant methanotrophic populations, together with their validation by directed experiments, could provide immensely useful information for policymakers and aid in instructing mitigation strategies directed at reducing methane efflux and increasing atmospheric methane uptake while also improving current climate models.

## Conclusions

Genome-scale metabolic models for aerobic methanotrophs have been useful for a variety of applications, including understanding basic metabolism under various nutrient conditions, formulating growth and nutrient strategies for gating carbon into targeted molecules of commercial value, and understanding metabolic and community dynamics between methanotrophs and associated microorganisms in ecosystems. *In silico* experiments performed with GEMs, such as ^13^C metabolic flux analysis, oxygen-to-carbon ratio experiments, or gene deletions/inclusions, save immense time and resources, as they can guide bench experiments. Despite methodological challenges and short-comings, the growing field of methanotroph GEM research with constructions of novel GEMs, the refinement of pre-existing GEMs, simultaneous development of genetic systems, incorporation of *-omics* information, combination of multiple GEMs, and construction of GEMs from MAGs are vastly expanding our understanding of methanotrophs in both single taxon and community contexts. With the rise of community GEMs, we hope to get a clearer view of the metabolic basis of inter-species interactions and their consequences for ecosystems. Applied in concert, GEM reconstruction, genetic tools, *−omics,* and physiological data will lead us more swiftly towards a viable single-carbon bioeconomy, biology-based methane mitigation strategies, and a better understanding of methanotrophs in the context of their environment and ecological interactions.

## Supplementary Material

SupplementaryS1_wrae102

## Data Availability

No data were generated in this study, and all data discussed is publicly available.
