# Supplementary material for: Leveraging genome-scale metabolic models to understand aerobic methanotrophs
Source: ISME J. 2024 Jun 11;18(1):wrae102. doi: 10.1093/ismejo/wrae102 (PMC11195481; doi:10.1093/ismejo/wrae102)
Supplement: SupplementaryS1_wrae102 [file supplementarys1_wrae102.docx]

Supplementary S1

A list of 71 marker genes used in constructing the tree is shown in Figure 1.

ADK, AICARFT_IMPCHas, ATP-synt, ATP-synt_A, Adenylsucc_synt, Chorismate_synt, EF_TS, Exonuc_VII_L, GrpE, Ham1p_like, IPPT, OSCP, PGK, Pept_tRNA_hydro, RBFA, RNA_pol_L, RNA_pol_Rpb6, RRF, RecO_C, Ribonuclease_P, Ribosom_S12_S23, Ribosomal_L1, Ribosomal_L13, Ribosomal_L14, Ribosomal_L16, Ribosomal_L17, Ribosomal_L18p, Ribosomal_L19, Ribosomal_L2, Ribosomal_L20, Ribosomal_L21p, Ribosomal_L22, Ribosomal_L23, Ribosomal_L27, Ribosomal_L27A, Ribosomal_L28, Ribosomal_L29, Ribosomal_L3, Ribosomal_L32p, Ribosomal_L35p, Ribosomal_L4, Ribosomal_L5, Ribosomal_L6, Ribosomal_L9_C, Ribosomal_S10, Ribosomal_S11, Ribosomal_S13, Ribosomal_S15, Ribosomal_S16, Ribosomal_S17, Ribosomal_S19, Ribosomal_S2, Ribosomal_S20p, Ribosomal_S3_C, Ribosomal_S6, Ribosomal_S7, Ribosomal_S8, Ribosomal_S9, RsfS, RuvX, SecE, SecG, SecY, SmpB, TsaE, UPF0054, YajC, eIF-1a, ribosomal_L24, tRNA-synt_1d, tRNA_m1G_MT
